# The Cas6e ribonuclease is not required for interference and adaptation by the *E. coli* type I-E CRISPR-Cas system

**DOI:** 10.1093/nar/gkv546

**Published:** 2015-05-26

**Authors:** Ekaterina Semenova, Konstantin Kuznedelov, Kirill A. Datsenko, Pierre M. Boudry, Ekaterina E. Savitskaya, Sofia Medvedeva, Natalia Beloglazova, Maria Logacheva, Alexander F. Yakunin, Konstantin Severinov

**Affiliations:** 1 Waksman Institute of Microbiology, Rutgers, the State University of New Jersey, Piscataway, NJ 08854, USA; 2 Department of Biological Sciences, Purdue University, West Lafayette, IN 47907, USA; 3 Laboratoire Pathogenèse des Bactéries Anaérobies, Institut Pasteur, Université Paris Diderot, Sorbonne Paris Cité, Cellule Pasteur, 25 rue du Dr Roux, 75724 Paris Cedex 15, France; 4 Skolkovo Institute of Science and Technology, Skolkovo 143025, Russia; 5 Institute of Molecular Genetics, Russian Academy of Sciences, Moscow 123182, Russia; 6 Institute of Gene Biology, Russian Academy of Sciences, Moscow 119334, Russia; 7 Department of Chemical Engineering and Applied Chemistry, University of Toronto, Toronto, Ontario M5S 3E5, Canada; 8 Belozersky Institute of Physico-Chemical Biology, Lomonosov Moscow State University, 119234 Moscow, Russia; 9 Pirogov Russian National Research Medical University, 117997 Moscow, Russia; 10 Peter the Great St. Petersburg Polytechnic University, St. Petersburg 195251, Russia

## Abstract

CRISPR-Cas are small RNA-based adaptive prokaryotic immunity systems protecting cells from foreign DNA or RNA. Type I CRISPR-Cas systems are composed of a multiprotein complex (Cascade) that, when bound to CRISPR RNA (crRNA), can recognize double-stranded DNA targets and recruit the Cas3 nuclease to destroy target-containing DNA. In the *Escherichia coli* type I-E CRISPR-Cas system, crRNAs are generated upon transcription of CRISPR arrays consisting of multiple palindromic repeats and intervening spacers through the function of Cas6e endoribonuclease, which cleaves at specific positions of repeat sequences of the CRISPR array transcript. Cas6e is also a component of Cascade. Here, we show that when mature unit-sized crRNAs are provided in a Cas6e-independent manner by transcription termination, the CRISPR-Cas system can function without Cas6e. The results should allow facile interrogation of various targets by type I-E CRISPR-Cas system in *E. coli* using unit-sized crRNAs generated by transcription.

## INTRODUCTION

CRISPR-Cas systems are common in prokaryotes and can provide small RNA-based adaptive immunity against mobile genetic elements (1,2). A CRISPR-Cas system consists of DNA loci with clustered regularly interspaced short palindromic repeats (CRISPR) and CRISPR associated (*cas*) genes. The CRISPR locus is composed of an array of identical or very similar repeats separated by spacers of common length but variable sequence. Some spacers originate from mobile genetic elements parasitizing on a prokaryotic host and the presence of such spacers can make the cell resistant to genetic parasites possessing sequences (referred to as protospacers) matching CRISPR spacers of the host (3).

It is convenient to divide CRISPR-Cas system function into three separate steps. The protective part of CRISPR-Cas mechanism during which foreign DNA is recognized and ultimately destroyed is referred to as ‘CRISPR interference’ (4). The part that leads to acquisition of spacers from foreign DNA into CRISPR loci is called ‘CRISPR adaptation’ (4). Another essential element of CRISPR-Cas function is the generation of unit-sized protective CRISPR RNAs (crRNAs) from the CRISPR locus transcript, referred to as ‘CRISPR expression’ (4). During this stage, pre-crRNA, a transcript of the entire CRISPR array, is generated and then processed with the help of Cas proteins or cellular RNases and small non-coding RNAs generating a set of crRNAs with common CRISPR repeat-derived 5′ and 3′ segments and central segments matching CRISPR spacers (5).

Of several diverse CRISPR-Cas systems known (6), the type I-E system of *Escherichia coli* is one of the best understood functionally. In this system, foreign DNA is recognized by a ribonucleoprotein complex composed of crRNA and Cascade—a ∼400 kDa protein assembly with subunit stoichiometry Cse1_1_Cse2_2_Cas7_6_Cas5_1_Cas6e_1_ (5,7). The Cascade-crRNA complex detects protospacer-containing DNA targets through initial interaction with a trinucleotide protospacer adjacent motif (PAM; preferred sequence AAG, (8,9)), recognized by the Cse1 subunit (7–8,10). The Cse1 and Cas5 are located at one end of the elongated Cascade structure and also interact with upstream repeat-derived 5′ handle of crRNA (10–13). The spacer-derived guide part of crRNA lies on the surface of the Cas7 hexamer, with most of its bases available for interactions with the target (11–13). Cas6e is located at the opposite side of the Cascade structure, interacting with downstream repeat-derived 3′ handle of crRNA (11–14). The Cascade-crRNA complex detects its targets with a functional PAM through complementary interactions between the crRNA guide element and a protospacer and induces localized DNA melting, generating R-loops (7). The Cas3 protein, an endonuclease and a helicase, is then recruited in the complex, leading to DNA target destruction (15,16).

The CRISPR adaptation process leads to acquisition of a spacer and an additional copy of CRISPR repeat. New sequences are added to the end of the CRISPR array that is proximal to promoter from which pre-crRNA transcription initiates (3). Two mechanisms of CRISPR adaptation in the *E. coli* subtype I-E system have been described. ‘Naïve’ or ‘non-primed’ adaptation requires just two most conserved Cas proteins, Cas1 and Cas2. When overexpressed, these proteins stimulate acquisition of new spacers in *E. coli* CRISPR array (17). Neither Cas1 nor Cas2 are required for CRISPR interference (5). During non-primed adaptation, spacers from both foreign (plasmid) and host DNAs are acquired, and both strands of donor DNA are used for spacer selection with equal efficiency. Only about 50% of spacers acquired during non-primed adaptation originate from protospacers with an AAG PAM required for interference (17). The second type of adaptation is referred to as ‘primed adaptation’ and is thought to require all type I-E Cas proteins (18,19). Primed adaptation is much more efficient than non-primed adaptation and also requires a crRNA with a guide element with some complementarity to target DNA. Residual recognition of the target by Cascade containing such crRNA leads to highly efficient acquisition of spacers from the DNA strand displaced by crRNA-protospacer interaction (18–20). As a result, more than 90% of spacers acquired during primed adaptation originate from this strand. Another hallmark of primed adaptation is a very strong bias toward the use of protospacers with functional AAG PAM as a source of spacers.

In *E. coli*, the protein components of Cascade are thought to be involved in crRNA maturation, DNA target binding and primed adaptation. The Cas6e protein is a Cascade component whose endoribonuclease activity is responsible for generation of unit-sized mature crRNAs by cleaving the pre-crRNA transcript in repeat-derived sequences (14). A single amino acid substitution of His^20^ for Ala that abolishes the endoribonuclease activity of Cas6e also abolishes both CRISPR interference and primed adaptation (5,18). However, it is still unclear if Cas6e is directly involved because both processes are dependent on the formation of mature crRNA. Cas6e can cleave pre-crRNA and generate unit-sized crRNAs even in the absence of other Cas proteins (5,21), raising a question whether pre-made crRNA can enter the Cascade and then function in CRISPR interference and primed adaptation. Indeed, a recent report showed that in an engineered archaeal CRISPR-Cas subtype I-B system, when crRNAs are generated by tRNA processing enzymes, the Cas6e homolog is dispensable for CRISPR interference (22). Here, we show that in the *E. coli* subtype I-E system, CRISPR interference and primed adaptation can proceed in the absence of Cas6e endonucleolytic activity or in fact without the Cas6e protein itself when mature crRNA is produced from engineered transcription units by means of factor-independent transcription termination. The results open the way for rapid CRISPR-Cas targeting of various host and foreign DNA sequences by crRNAs generated by transcription termination and demonstrate that the multisubunit subtype I-E CRISPR-Cas system can be reduced in complexity, making it potentially attractive for biotechnological applications.

## MATERIALS AND METHODS

### Plasmid and strain construction

Plasmids and strains used in this study are provided in Supplementary Tables S1 and S2, respectively. Plasmids encoding unit-sized crRNA were constructed by cloning synthetic dsDNA duplex fragments (gBlocks from IDT Inc.) containing T7 A1 or *trp* promoter and crRNA coding sequences followed by (T)_8_ track into EcoNI and KpnI sites of pACYCDuet-1 vector (Novagen). Three T7 A1 promoter containing plasmids were prepared: pG8_crRNA contains g8 spacer sequence targeting M13 phage DNA (genome positions 1358–1389); the plasmid pSpT5_crRNA contains T5 spacer sequence targeting T5 phage DNA (genome positions 1869–1900, complementary strand); the plasmid pSpλ_crRNA contains λ spacer sequence targeting phage λ DNA (genome positions 29798–29829, complementary strand). Spacer sequences were chosen based on the presence of functional PAM sequences upstream of corresponding protospacers in phage genomes. In plasmid pG8trp_crRNA, the g8 crRNA coding sequence was cloned under control of the *E. coli trp* operon promoter.

Mutation C1T at the first position of the spacer was introduced into pG8_crRNA by QuickChange Site-Directed Mutagenesis Kit (Stratagene) according to manufacturer's protocol, creating plasmid pG8_crRNA_C1T. Plasmids encoding Cascade subunits and CRISPR cassette containing multiple g8 spacers are described in (5,8–9,23). The pT7Blue-based plasmids carrying a 209-bp M13 fragment with the g8 protospacer (genome positions 1311–1519) with or without an escape mutation C1T at the first position of the protospacer is described in (18).

Strains producing Cascade proteins for *in vitro* experiments are described in (18,23). Strains KD390, KD477 and KD599 created for this work are derivatives of KD263 (18) and were constructed using the previously published Red recombinase protocol (24).

### RNA extraction and Northern blotting

The procedure was performed essentially as described (25). Total RNA was isolated from 2 ml of *E. coli* cells grown until OD_600_ reached 1 and lysed by 5-min treatment using Max Bacterial Enhancement Reagent with subsequent purification by the TRIzol reagent (Invitrogen). RNAs bound to Cascade were extracted from purified complexes by TRIzol reagent. 10 μg of total RNAs or 20–40 ng of Cascade-purified RNAs were separated on a denaturing 8 M urea - 12% polyacrylamide gel and electrophoretically transferred to Hybond-XL membrane (GE Healthcare) using a Mini Trans-Blot Electrophoretic Transfer Cell (Bio-Rad). The membrane was dried and then UV cross-linked. ExpHyb hybridization solution (Clontech) was used for hybridization according to manufacturer's instructions for 1 h at 37°C with ^32^P-end labeled g8-spacer specific probe (5′-GCGGGATCGTCACCCTCAGCAGCGAAAGACAG-3′).

### Cascade purification

Cascade or Cascade subcomplexes lacking Cse1, Cas6e, or Cas6e and Cse1 were prepared from cells co-expressing appropriate *cas* genes (with or without a source of crRNA and were one-step affinity-purified on *Strep*-Tactin® column (IBA) using the N-terminal Strep-tag attached to the Cse2 subunit (23) according to manufacturer's protocol. N-terminal 6His-tagged Cse1 protein was IMAC purified from *E. coli* KD418 strain and further purified using gel-filtration on Superdex 200 HiLoad 16/60 column (Amersham Biosciences) equilibrated with 20 mM HEPES-K buffer (pH 7.5) containing 150 mM NaCl. We also purified two Cascade forms (with and without Cas6e) from cells lacking any source of crRNA using a protocol published earlier (23).

### CRISPR interference and adaptation assays

*E. coli* strain carrying a genomic CRISPR array with g8 spacer (KD263, (18)) or strains KD390, KD477 and KD599 which harbor only a CRISPR repeat in their genome and which were supplemented with plasmids encoding unit-sized crRNA were used to determine cell sensitivity to phage infection by a spot test method as described (8). To perform crRNA expression from the *trp* promoter, KD390 cells transformed with the plasmid pG8trp_crRNA were grown in minimal medium M9 supplemented with 0.4% glycerol and 34 μg/ml chloramphenicol at 37°C until OD_660 nm_ reached 0.5, then 3-β-indoleacrylic acid (IAA) was added to the final concentration 10 μg/ml for crRNA expression; IPTG and arabinose were added to 1 mM each to induce *cas* genes expression. After 4 h induction the cells were used for plating and spot test with the M13 phage. Efficiency of plaquing was calculated as a ratio of the number of plaques formed on a lawn of tested cells to the number of plaques on sensitive (non-targeting, KD390) cell lawn. For each host-phage variant, plaquing efficiency was determined in at least three independent experiments. Escape phage plaques were analyzed by sequencing through the g8 protospacer region as described in (8). For plasmid transformation efficiency assays, competent pre-induced cells were prepared and electroporated with 10 ng of DNA from protospacer carrying plasmid (pG8, pG8_mut or pT7blue as a control). Transformation efficiency was determined as ampicillin-resistant colony numbers per μg DNA. The experiments were repeated at least three times.

To monitor CRISPR adaptation, KD263 cells and cells (KD390, KD477, KD599) carrying the pG8_crRNA plasmid were transformed with protospacer carrying plasmid pG8, individual colonies were selected, and grown overnight at 37°C in LB broth supplemented with 100 μg/ml ampicillin and 34 μg/ml chloramphenicol where needed. Aliquots of cultures were diluted 200-fold with fresh LB broth without ampicillin and supplemented with IPTG and arabinose to the final concentration 1 mM each. The cultures were grown at 37°C overnight. To monitor spacer acquisition, 1 μl of culture was added to 20 μl PCR reaction and amplification was performed with primers: Ec_LDR-F (5′-AAGGTTGGTGGGTTGTTTTTATGG-3′) and Ec_minR (5′-CGAAGGCGTCTTGATGGGTTTG-3′). PCR products corresponding to expanded CRISPR cassettes were gel purified using QIAquick Gel Extraction Kit (QIAGEN) and sequenced with MiSeq Illumina System at Moscow State University Genomics facility.

### *In vitro* transcription

Double-stranded DNA fragment containing unit-sized g8 crRNA sequence flanked upstream with the T7 A1 promoter and downstream with (T)_8_ track was prepared by PCR amplification from pG8_crRNA plasmid. The resulting fragment had coordinates −96/+149 with respect to the transcription start site located at +1. To allow open complex formation, 3.3 nM transcription template was incubated with 100 nM of *E. coli* σ^70^ RNA polymerase holoenzyme in 10 μl of transcription buffer containing 35 mM Tris-HCl, 70 mM NaCl, 7 mM MgCl_2_, 0.7 mg/ml BSA, (pH 7.9 at 25°C) for 10 min at 37°C. Transcription was initiated by the addition of NTPs (200 μM ATP and GTP, 100 μM CTP, and 10 μM of UTP). 5 μCi of [α-^32^P]-UTP (3000 Ci/mmol) was also added. After 5-min incubation at 37°C, reaction was terminated by the addition of equal volume of formamide. RNA products were separated by 20% polyacrylamide gel electrophoresis (PAGE) at denaturing condition (8 M urea) and analyzed by autoradiography.

### KMnO_4_ probing

The target g8 dsDNA (209 bp) was PCR amplified from pG8 plasmid containing g8 protospacer (Supplementary Table S1). The 5′ ends of the target ds-DNA fragment were labeled with ^32^P using T4 polynucleotide kinase for 30 min at 37°C (5–10 pmoles of 5′ termini in 30 μl reaction mixture containing 70 mM Tris-HCl (pH 7.6), 10 mM MgCl_2_, 5 mM DTT, 10 pmoles [γ-^32^P]-ATP (6000 Ci/mmol)) and 10 units of T4 polynucleotide kinase (New England Biolabs). ^32^P-labeled DNA fragments were purified on micro Bio-Spin^TM^ chromatography columns packed with Bio-Gel P-6 (BIO-RAD). Oxidative modification by KMnO_4_ was performed with 5-nM-labeled target DNA, 0.2–0.4 μM of Cascade complex and, where necessary, 0.5–1 μM crRNA in 10 μl binding buffer (40 mM Tris-HCl, pH 8.0, 50 mM NaCl). When chemically synthesized crRNA was added, Cascade and crRNA were first mixed and incubated at 37°C for 5 min to allow Cascade-crRNA complex formation. Target DNA was added to Cascade-crRNA complex and incubated at 37°C for 25–30 min. The probing reaction was initiated by adding KMnO_4_ to a final concentration of 2.5 mM. Reactions were incubated for 15 s at 37°C, quenched by the addition 10 μl of 1% β-mercaptoethanol and 5 μg of calf thymus DNA in 50 μl of 10 mM Tris-HCl (pH 8.5) and extracted with phenol-chloroform mixture, followed by ethanol precipitation. DNA pellets were dissolved in 100 μl of freshly prepared 1 M piperidine and heated in dry-bath at 95°C for 20 min. Piperidine was removed by chloroform extraction and DNA was ethanol precipitated. Pellets were dissolved in 10 μl of 10 mM Tris-HCl (pH 8.5) and supplemented with 15 μl of formamide loading buffer with Bromophenol Blue and Xylene Cyanol. Samples were separated by electrophoresis in 8% denaturing polyacrylmide gels containing urea. After electrophoresis, the gel was fixed in 10% acetic acid, dried and exposed to a phosphorus screen overnight. The images were scanned using PhosphorImager (Molecular Dynamics) and analyzed using ImageQuantMac v 1.2.

### High throughput sequence data analysis

Raw sequencing data were analyzed using ShortRead and BioStrings (26) packages. Illumina-sequencing reads were filtered for quality scores of ≥20 and reads containing two repeats (with up to two mismatches) were selected. Reads that contained 33-bp sequences between two CRISPR repeats were next selected and considered as spacers. Spacers were next mapped on the pG8 plasmid with no mismatches allowed. R scripts were used for spacers statistics and Circos (27) was used for graphical representation of the data.

## RESULTS

### *In vivo* CRISPR interference by unit-sized crRNA generated without Cas6e processing

**Figure 1.**
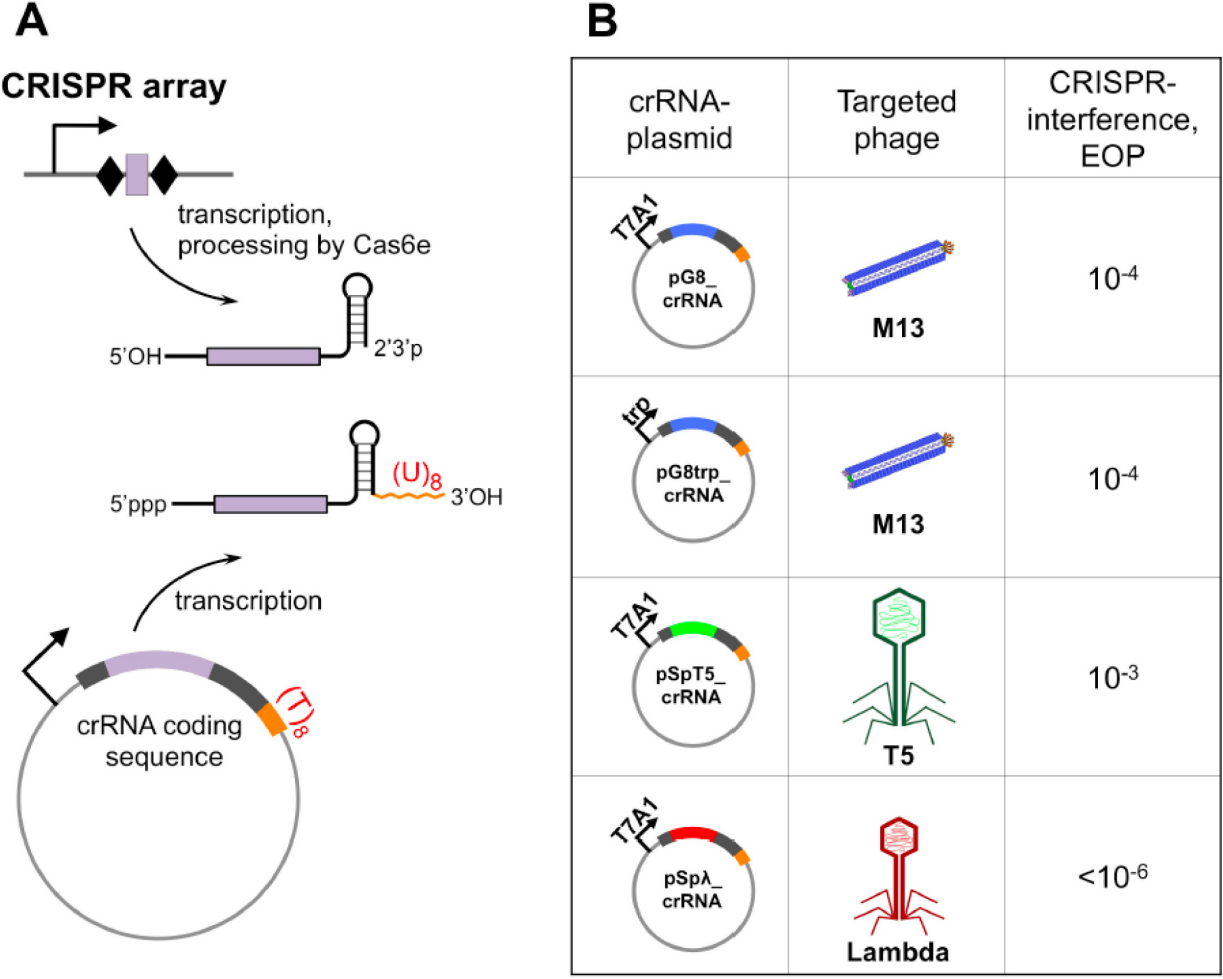
Cells harboring plasmids expressing crRNA-like transcription units become resistant to phage infection. **(A)**. Two ways of crRNA generation — by Cas6e-mediated endonucleolytic processing of pre-crRNA transcript of CRISPR array or by transcription termination on an intrinsic transcription terminator build upon the hairpin structure formed by the 3′-terminal repeat — are schematically shown. (**B)**. CRISPR interference, calculated as efficiency of plaquing (EOP) of indicated phages on indicated cells lawns is shown. An EOP of 1 indicates that the number of plaques formed on lawns of cells with induced *cas* gene expression was the same as the number of plaques on lawns of cells which did not express any crRNA. For every non-cognate cell-phage combination, an EOP of 1 was observed.

To generate unit-sized crRNA independently of Cas6e endoribonucleolytic activity, DNA fragments corresponding to three mature crRNAs were cloned on plasmids under control of a strong σ^70^ RNA polymerase-dependent T7 A1 promoter (Figure 1A). The point of transcription start site coincided with the 5′ end of mature crRNA. Following the 7-bp sequence of the 5′ crRNA handle, 33 bp DNA fragments matching sequences adjacent to functional PAMs in bacteriophages M13, T5 or λ were located. The M13 sequence was identical to the previously characterized g8 spacer capable of interference in the context of complete *E. coli* type I-E CRISPR-Cas system (8,18). To generate crRNA 3′ end, the palindromic sequence of CRISPR repeat located downstream of the spacer part of the engineered transcription unit was fused to a run of eight thymidine residues, resulting in a structure mimicking a factor-independent transcription terminator (28). A plasmid pG8trp_crRNA, from which g8 crRNA was transcribed from the inducible *E. coli trp* promoter was also created.

Transcription termination is expected to result in a ∼70-nt long transcript containing a 5′ triphosphate moiety (not present in mature crRNA) and a 3′ terminal repeat hairpin followed by a U-tract containing up to eight uridine residues (Figure 1A). Indeed, an *in vitro* transcription experiment with *E. coli* σ^70^ RNA polymerase holoenzyme and a DNA fragment containing the T7 A1 promoter and initial transcribed part matching g8 crRNA revealed robust transcription termination at the U-tract (Supplementary Figure S1).

Plasmids containing the resulting engineered transcription units were introduced in *E. coli* KD390 cells, which contain *cas* genes under the control of two inducible promoters *lac*UV5 (*cas3*) and *ara*Bp8 (*cse1cse2cas7cas5cas1cas2*) (18). Genomic CRISPR arrays have been deleted from the KD390 genome with only one repeat and complete leader sequence of the CRISPR 2_1 array remaining. We next determined if KD390 cells harboring these plasmids and lacking other sources of crRNA were capable of CRISPR interference. To this end, the efficiency of plaquе formation by the wild-type M13, T5, and λ phages on lawns of induced KD390 harboring crRNA plasmids was determined. The results are schematically shown in Figure 1B. As can be seen, strong protection from phage infection was observed. The effect was specific, since cells were protected only from ‘cognate’ phages, while efficiency of infection with non-cognate phages equaled 1. To observe protection of cells with constitutive T7 A1 promoter-driven crRNA expressing plasmids from phage infection only induction of *cas* genes was necessary. In contrast, for cells transformed with the pG8trp_crRNA plasmid where the crRNA transcription unit was under the control of *trp* promoter, induction of transcription from this promoter by IAA was also required (see 'Materials and Methods' section).

**Figure 2.**
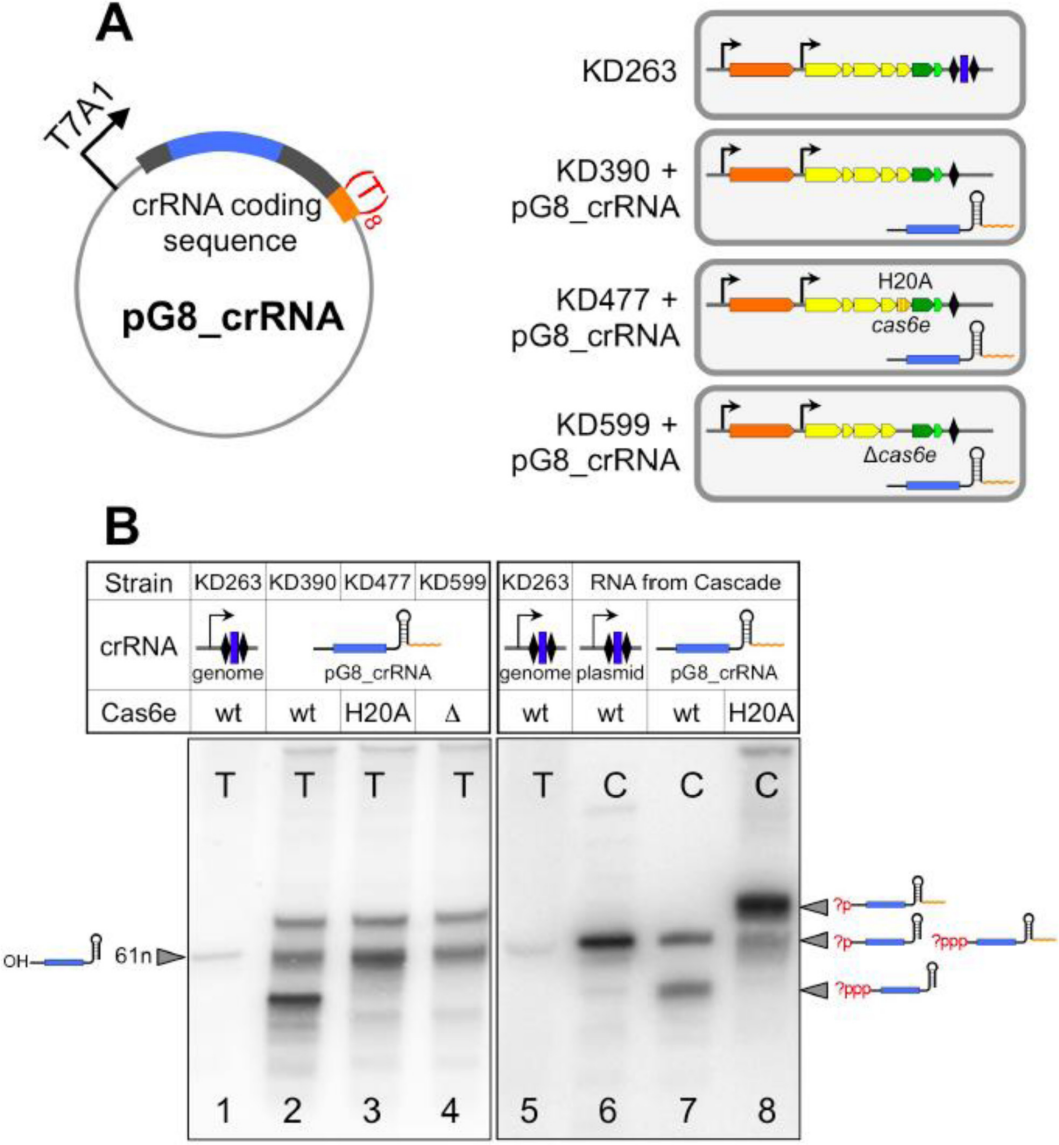
Generation of unit-sized crRNA by transcription termination *in vivo*. (**A**). The pG8_crRNA plasmid designed to express crRNA containing a spacer (shown in blue) targeting the M13 phage and *E. coli* strains used in this work are schematically presented. Colored arrows represent *cas3* (orange), *cse1cse2cas7cas5cas6e* (yellow) and *cas1* and *cas2* (dark and light green, respectively). Transcription of *cas3* and *cse1cse2cas7cas5cas6ecas1cas2* operon is driven by inducible promoters (shown as thin black arrows). Rhombi indicate CRISPR repeats, a blue rectangle in KD263 represents the g8 spacer. The crRNA transcript produced from the pG8_crRNA plasmid is indicated. (**B**). Northern blot analysis of RNA prepared from cells shown in panel A and hybridizing to g8 spacer probe (lanes 1–5) or from Cascade purified from indicated cells (lanes 6–8). Likely structures of RNAs migrating as separate bands on the gel are schematically shown on the right. Question marks indicate uncertainty about the structure of 5′ ends of crRNA transcripts. T: total RNA, C: RNA extracted from Cascade.

The protection afforded by the pG8_crRNA plasmid from the M13 infection was investigated further, as it is governed by a well-characterized interaction of g8 crRNA with its protospacer. First, we wished to demonstrate the generation of unit-sized g8 crRNAs from the pG8_crRNA plasmid *in vivo*. A functional Cas6e protein, when present, may recognize the 3′ terminal CRISPR repeat-derived part of plasmid-borne crRNA and process it. In this case, a crRNA corresponding to normal 61-nt crRNA but bearing a 5′-terminal triphosphate should be produced. The 5′ triphosphate of the crRNA transcript can be processed by cellular phosphodiesterases or phosphatases producing monophosphorylated or dephosphorylated transcripts (29). In addition to KD390 harboring pG8_crRNA, the pG8_crRNA plasmid was also introduced in KD477, a KD390 variant carrying a non-functional *cas6e*^H20A^ allele (Figure 2A; hereafter referred to as ‘H20A’). As a control, RNA purified from KD263 cells containing inducible *cas* genes and genomic CRISPR cassette with g8 spacer ((18), Figure 2A) was used. As expected, a single g8 probe-hybridizing band corresponding to 61-nt mature g8 crRNA was detected in KD263 cells (Figure 2B, lane 1 and Supplementary Figure S2). In RNA from KD390 cells harboring pG8_crRNA, a major g8 probe-hybridizing band that migrated faster than mature 61-nt g8 crRNA was detected (Figure 2B, lane 2). A less intense diffuse probe-hybridizing band had a mobility matching that of g8 crRNA, while another band of similar intensity had lower mobility on the gel. The major faster migrating crRNA-like product present in KD390 was not observed in H20A strain harboring pG8_crRNA, while the other two bands hybridizing with the g8 spacer probe were present (Figure 2B, lane 3). It therefore follows that the faster moving band in KD390 harboring pG8_crRNA is a product of nucleolytic processing by Cas6e.

We next purified Cascade from induced cells harboring pG8_crRNA through an affinity tag located on the Cse2 subunit and repeated the northern blot experiment with RNA from Cascade. As a control, Cascade purified from *E. coli* cells harboring a plasmid-borne T7 RNA polymerase promoter-driven CRISPR cassette containing multiple g8 spacers (8,9) was used. Elsewhere, it is shown that such Cascade is loaded with correctly processed mature crRNA (8,9). Control Cascade contained RNA with mobility matching that of mature crRNA present in KD263 cells (Figure 2B, compare lanes 5 and 6), as expected. Cascade purified from pG8_crRNA-containing cells with wild-type *cas* genes contained two g8-hybridizing RNAs, one with mobility matching that of mature crRNA and another with faster mobility (Figure 2B, lane 7). Cascade from cells with catalytically inactive Cas6е was purified in the same yield as wild-type Cascade and also contained two RNA species, a minor one whose mobility matched that of mature crRNA and a much more abundant slower migrating RNA (Figure 2B, lane 8). Bands present in RNA from cells producing non-functional Cas6е endonuclease mutant should contain a hairpin formed by the 3′ terminal repeat and additional uridine residues at the 3′ terminus produced during transcription termination. The 5′-terminal triphosphate present on *de novo* initiated transcript is known to increase electrophoretic mobility of RNA (30) and is absent from mature *E. coli* crRNA (5,7). We therefore propose that the presence of two distinct crRNA bands in Cascade preparations from cells where crRNA is generated by transcription termination may be due to the presence of fast moving species with 5′-triphosphate and a slower moving species with 5′-monophosphate. The presence of 5′-hydroxyl is not supported by results of T4 kinase labeling, which showed that only mature crRNA and a chemically synthesized 61-nt long g8 crRNA were efficiently labeled with this enzyme (Supplementary Figure S3). We note that assignments for crRNA-like bands shown in Figure 2B are tentative; however, interpretation of functional data presented below does not depend on exact transcript assignments.

The results of northern blot analysis clearly show that transcription terminator based on CRISPR repeat hairpin is functional *in vivo* and cells containing pG8_crRNA produce crRNA-like transcripts some of which enter the Cascade complex. We note that the slow-moving crRNA variant is absent from Cascade purified from cells with wild-type *cas6e*, even though it is present in cells (compare Figure 2B, lanes 2 and 7). The longer crRNA species produced by transcription termination is likely cleaved by Cas6e endonuclease once it becomes part of the wild-type Cascade complex. On the other hand, when Cas6е endonuclease is inactivated the cleavage generating proper 3′ crRNA end cannot occur but the primary crRNA transcript still enters the Cascade complex.

**Figure 3.**
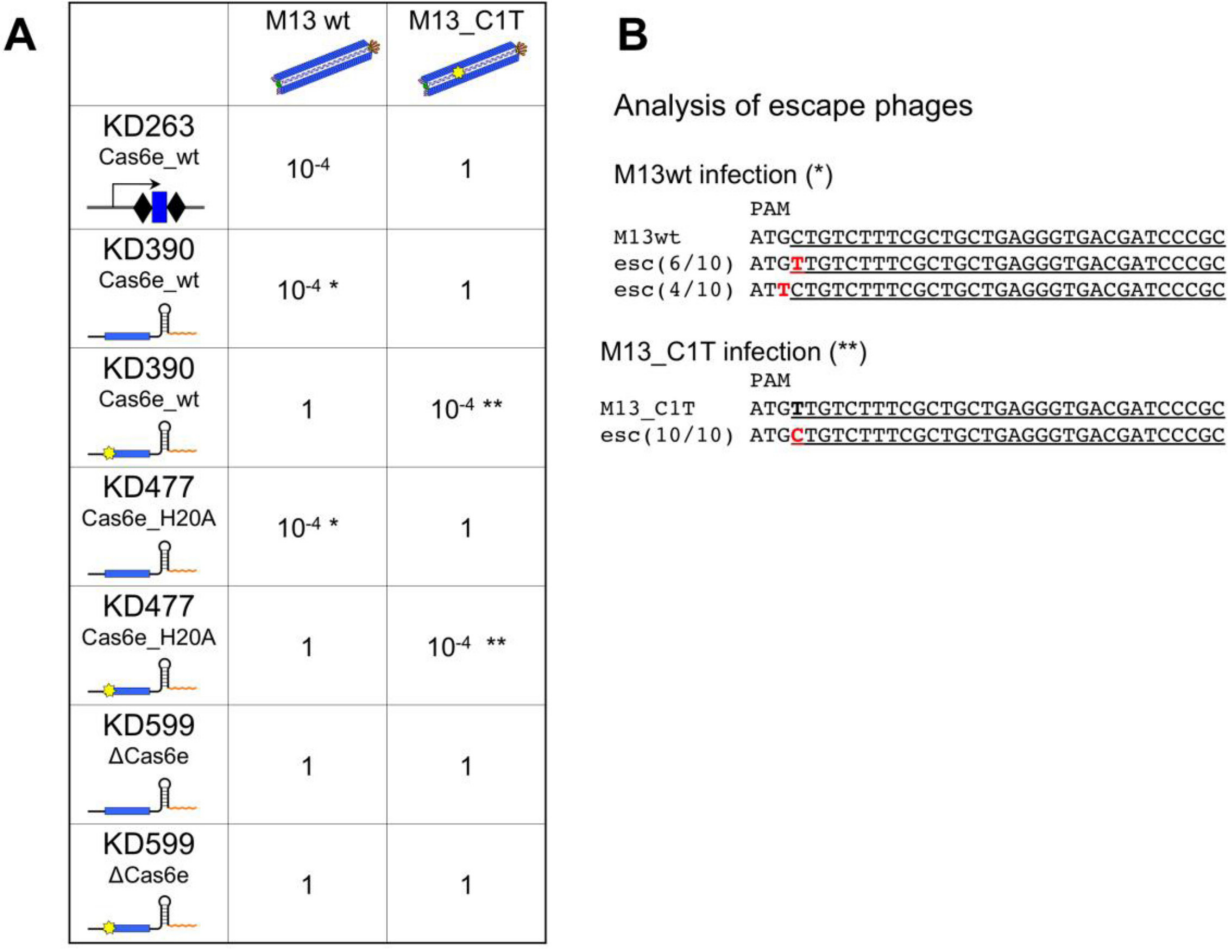
CRISPR interference with M13 phage infection by cells producing unit-sized crRNA through transcription termination. (**A**). Efficiency of plaquing of wild-type M13 and an escape mutant harboring a C to T substitution at the first position of the g8 protospacer (indicated with a yellow star) on indicated cells is shown. Asterisk or double asterisks show that phages from rare plaques that were formed on tested induced cell lawns contained mutations in the protospacer that rendered CRISPR interference inactive (shown in panel **B**).

The results of phage sensitivity tests (Figure 1B) strongly suggest that unit-sized crRNAs produced by transcription termination are capable of interference. To further prove that the observed interference is specific, efficiency of plaquе formation by the wild-type M13 phage on lawns of induced KD390 harboring pG8_crRNA was determined using both the wild-type, and the mutant M13g8_C1T phage harboring a point mutation at the first position of g8 protospacer (C1T), which renders CRISPR interference inactive (i.e. leads to an ‘escape’ phenotype) in the context of complete system (8), was tested. As can be seen from Figure 3A, cells harboring pG8_crRNA interfered with phage infection as efficiently as KD263 cells that produced interfering g8 crRNA from a genomic CRISPR array (at least four orders of magnitude decreased infection efficiency). The growth of escape phage was unaffected. Ten phage isolates from rare plaques formed on lawns of KD390 harboring pG8_crRNA were analyzed by sequencing through the protospacer region. Six phages had mutations in the first position of the seed, while remaining in the PAM of the g8 protospacer (Figure 3B). Both mutations were previously shown to lead to escape phenotype in the context of interference by complete CRISPR-Cas system (8,18). We therefore conclude that cells expressing unit-sized crRNA mutations restrict phage growth due to CRISPR-Cas interference.

An additional crRNA expressing plasmid, pG8_crRNA_C1T, containing a C to T substitution at the +1 position of the spacer was created. When KD390 cells harboring this plasmid were tested in phage infection assay, the pattern of sensitivity to the wild-type and C1T escape phage was reversed. Cells carrying pG8_crRNA_C1T interfered with the mutant phage infection but were efficiently infected by the wild-type phage (Figure 3A). From the rare plaques formed after infection of KD390 cells harboring the pG8_crRNA_C1T with M13g8_C1T, 10 plaques were randomly picked up and phage DNA sequenced through the protospacer region. In all cases, mutations at the first protospacer position that reversed the sequence to the wild-type state were detected (Figure 3B). We conclude that crRNA produced by transcription termination rather than from pre-crRNA is capable of causing specific CRISPR interference in *E. coli* when Cas proteins are present in the cell.

We next determined if Cas6е endoribonuclease activity, normally essential for CRISPR interference (4), is also required in cells in which crRNA is produced by transcript termination. Cultures of H20A cells carrying pG8_crRNA were as well protected from the wild-type but not M13g8_C1T phage infection, as KD390 cells (Figure 3A). Conversely, when H20A cells carried pG8_crRNA_C1T, they were protected from M13g8_C1T but not wild-type phage infection (Figure 3A). Thus, the Cas6e endonuclease activity is not required for CRISPR interference when a source of processed crRNA is available.

KD599 (‘Δcas6e’), a derivative of KD390 entirely lacking the *cas6е* gene was constructed and transformed with pG8_crRNA. Northern blot analysis showed that the pattern of g8 probe hybridizing RNAs in Δcas6e cells harboring pG8_crRNA was the same as in H20A cells (Figure 2B, lane 4). Repeated attempts to purify Cascade from these cells resulted in very low yields and no RNA hybridizing to g8 probe could be detected (data not shown), suggesting that crRNA association with Cascade is stabilized by the presence of Cas6е. The Δcas6e cells transformed with pG8_crRNA or pG8_crRNA _C1T were equally sensitive to wild-type or escape phage infection (Figure 3A) suggesting either that Cas6е protein itself is required for CRISPR interference or the phage resistance assay is not sensitive enough when the amount of crRNA-bound Cascade is reduced.

**Figure 4.**
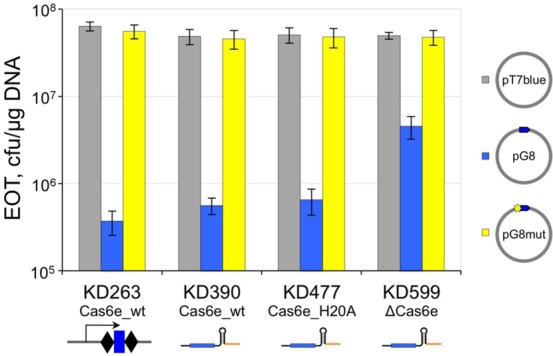
Cells producing unit-sized crRNA through transcription termination interfere with plasmid transformation. Efficiency of transformation (EOT) of three plasmids—pG8 containing a wild-type g8 protospacer and a functional PAM (blue bars), pG8mut, containing g8 protospacer with a C to T substitution at position +1 (yellow bars), and the pT7 blue vector (gray bars)—into indicated induced cells is presented. The height of the bars represents mean value obtained in three independent experiments and standard deviations are shown.

We next tested CRISPR interference using a plasmid transformation assay. Cells harboring pG8_crRNA were transformed with compatible ampicillin-resistance plasmids with g8 protospacer-PAM, an escape version of this protospacer, or an empty vector with no g8 sequence and efficiency of transformation (EOT) was determined. Transformation of KD263 cells with same plasmids was performed as control. As can be seen from Figure 4, EOT of KD263 was reduced ∼100-fold when a functional protospacer PAM was present on the plasmid. The same decrease was observed in KD390 and H20A cells carrying pG8_crRNA, indicating, in agreement with phage infection data, that these cells are capable of CRISPR interference. EOT of plasmid containing wild-type protospacer in Δcas6e cells harboring pG8_crRNA was decreased 10-fold. The effect was specific, since plasmid containing an escape mutation in the protospacer transformed as efficiently as the empty vector control (Figure 4). It therefore follows that in *E. coli* CRISPR interference can occur in the absence of Cas6е, albeit with decreased efficiency, when unit-sized crRNA is produced by transcription termination, bypassing the normal pre-crRNA processing step.

Comparison of CRISPR interference tests results in bacteriophage infection and plasmid transformation assays reveal that the latter assay is more sensitive. A similar conclusion was reached when studying the effects of alterations of PAM sequences on interference by the *E. coli* subtype I-E CRISPR system (9). Presumably, attenuated interference in the absence of Cas6e (or in the presence of suboptimal PAM sequences in DNA targets) is masked during formation of viral plaques, which result from multiple infectious cycles following the initial infection, but is more apparent during bacterial colony formation from a single transformed cell.

### *In vitro* target recognition by Cascade lacking Cas6e

**Figure 5.**
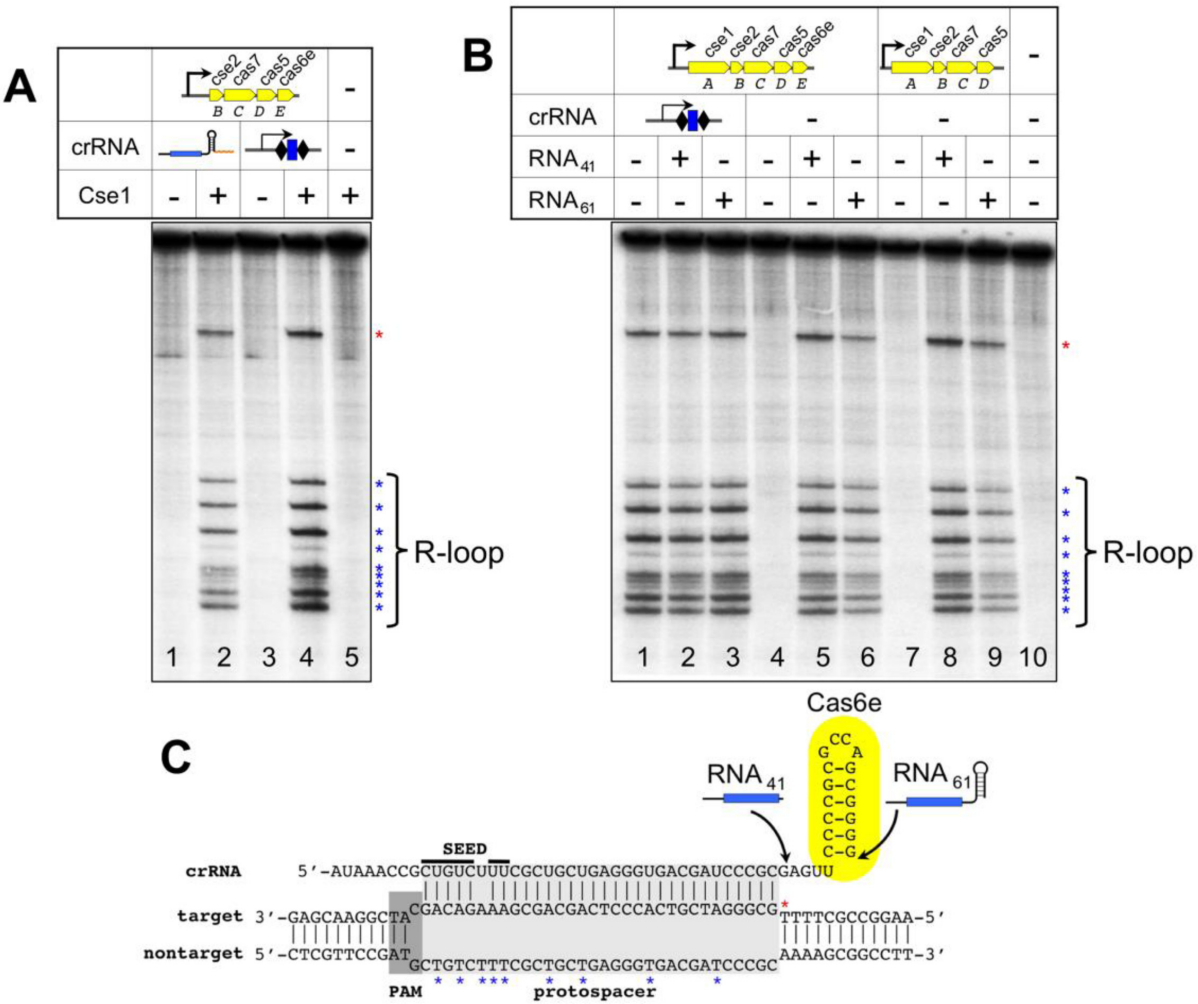
Formation of R-loops *in vitro*. (**A**) Cascade was affinity purified from *E. coli* cells lacking genomic CRISPR arrays, expressing all Cascade subunits but Cse1 and harboring the pG8_crRNA plasmid producing unit-sized crRNA transcript (lanes 1 and 2), or a plasmid containing a CRISPR array with multiply repeated g8 spacer (lanes 3 and 4). Where indicated, Cascade preparations were supplemented with recombinant Cse1, combined with terminally labeled double-stranded DNA fragment containing the g8 protospacer and functional PAM and subjected to potassium permanganate probing. Reaction products were resolved by denaturing urea gel and revealed by phosphoimagery. (**B**) Cascade was purified from cells lacking any source of crRNA (lanes 4–6) or cells that lacked both the crRNA source and the *cas6e* gene (lanes 7–9). In lanes 1–3, control Cascade purified from cells harboring a plasmid containing a CRISPR array with multiply repeated g8 spacer was used. Where indicated, Cascade preparations were supplemented with chemically synthesized g8 crRNA (RNA_61_) or its truncated variant lacking the 3′ handle (RNA_41_) and used in permanganate probing reactions set up and analyzed as described for panel A. (**C**) Summary or permanganate probing. The R-loop structure formed on the g8 protospacer target DNA is shown. Asterisks indicate permanganate-sensitive thymine positions in the R-loop complex. The end points of synthetic crRNA molecules used are indicated. The 3′ repeat handle is shown within a yellow oval that represents a tightly bound Cas6e molecule.

To further investigate specificity of the Cas6е-less Cascade toward target DNA binding and support *in vivo* plasmid interference data, we purified Cascade from *E. coli* cells co-overexpressing pG8_crRNA, which was the only source of crRNA, and all Cascade components but Cse1 (Supplementary Figure S4). As a control, Cascade purified from *E. coli* cells harboring a plasmid-borne T7 RNA polymerase promoter-driven CRISPR cassette containing multiple g8 spacers was used (8,9). Such Cascade, when supplemented with recombinant Cse1, is able to specifically locate DNA targets (31). We therefore supplemented both Cascade complexes with recombinant Cse1 and tested them for ability to form R-loops on a double-stranded DNA target containing the g8 protospacer and functional PAM using permanganate probing, which reports on the presence of thymidine residues in single-stranded form. R-loop formed by Cascade with crRNA produced by transcription termination was indistinguishable from that formed by Cascade containing native crRNA (Figure 5A, compare lanes 2 and 4).

As already mentioned, Cascade purified from cells lacking Cas6e does not contain crRNA detectable by northern blotting. We therefore purified Cascade from cells lacking any source of crRNA and overexpressing all Cascade subunits or just Cse1, Cse2, Cas7 and Cas5 through an affinity tag on Cse1 ((23) and Supplementary Figure S5). Both Cascade preparations were supplemented with chemically synthesized g8 crRNA, a 61-nt long variant matching mature crRNA or a 41-nt long version that lacks all but one nucleotide 3′ terminal to the spacer. Both RNAs bind crRNA-free Cascade *in vitro* and the resulting complex forms R-loops with complementary double-stranded targets (23). Cascade without Cas6e, when supplemented with synthetic full-sized crRNA (RNA_61_) or minimalized RNA_41_, also recognized target DNA and formed R-loops that were indistinguishable from those formed by complete Cascade by permanganate probing (Figure 5B, compare lanes 5 and 6 with lanes 8 and 9). Unlike Cascade purified from cells expressing g8 crRNA, both crRNA-free Cascade preparations depended on exogenously added synthetic crRNAs for R-loop formation with the DNA target (Figure 5B, compare lane 1 with lanes 4 and 7).

Overall, the results of *in vitro* analysis confirm that Cascade carrying a unit-length crRNA produced by transcription termination rather than Cas6e-dependent processing and/or lacking Cas6e is competent for target recognition and formation of apparently complete R-loops with target DNA.

### Cas6e-independent primed adaptation

**Figure 6.**
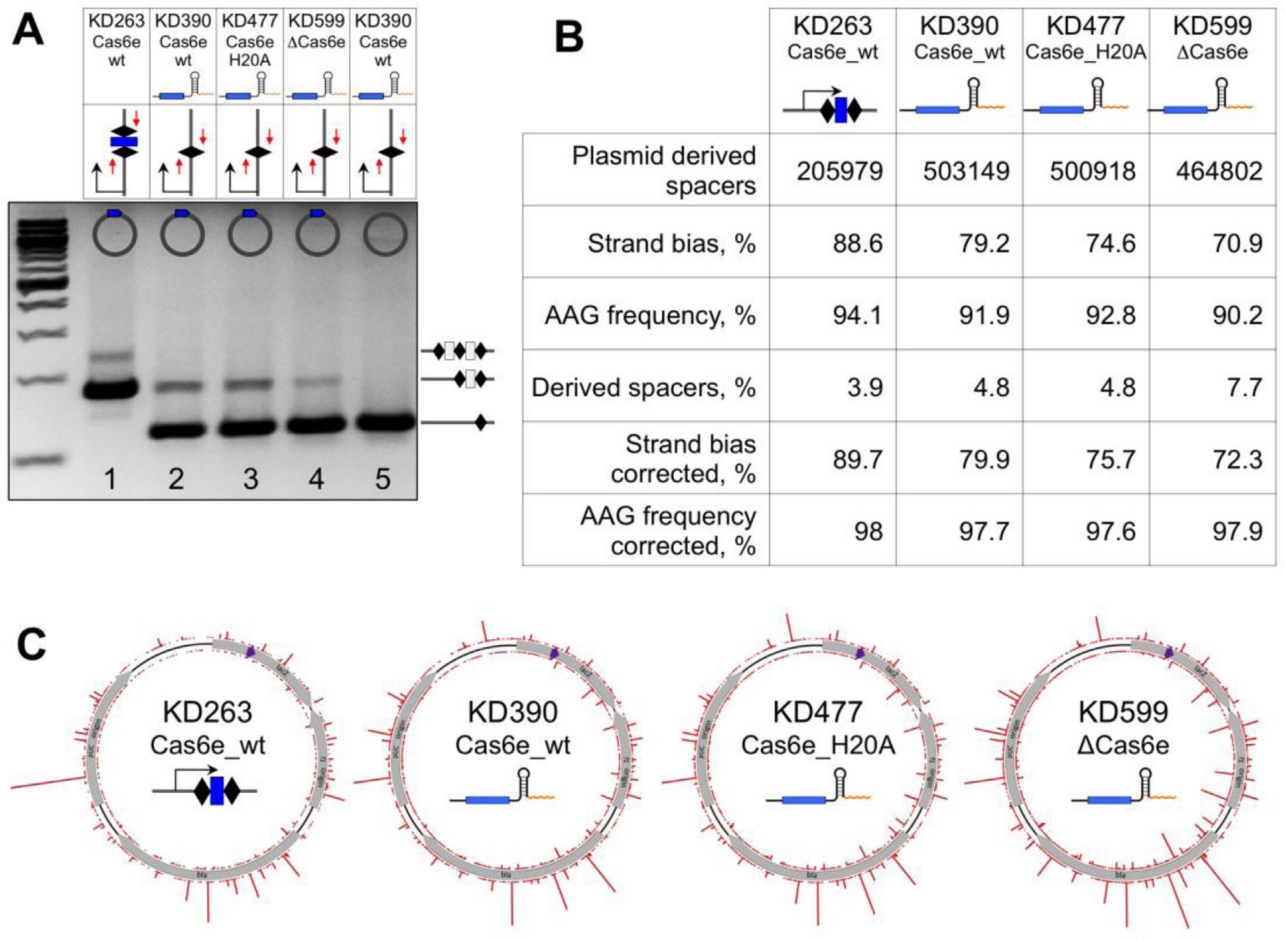
Primed CRISPR adaptation in cells producing unit-sized crRNA transcripts with or without Cas6e. (**A**) Results of primed adaptation experiment (see ‘Materials and Methods’ section) with indicated cells are shown. The leader-proximal end of CRISPR cassette was amplified using an appropriate primer pair, amplification products were separated by agarose gel electrophoresis and revealed by ethidium bromide staining. **(B)** DNA fragments corresponding to expanded CRISPR cassettes shown in (A) were subjected to Illumina sequencing. Statistics for reads corresponding to spacers derived from priming protospacer-containing plasmid is presented. ‘Derived spacers’ refers to spacers that originate from recognition of protospacers with correct AAG PAM but are either excised from target DNA with short shifts in the upstream or downstream direction or are inverted during acquisition in CRISPR array. (**C**) Mapping of spacers acquired in (A) on the priming protospacer plasmid is schematically shown. The position of the priming protospacer (purple triangle) as well as known features of the plasmid are shown. The heights of red bars indicate the efficiency (number of times) a spacer arising from a protospacer in this position was observed. Red bars protruding inside and outside of circles indicating the protospacer plasmid show spacers derived from different strands of DNA.

Cells carrying a *cas6e*^H20A^ allele and expressing crRNA from natural CRISPR arrays are incapable of primed adaptation (18). We followed CRISPR adaptation in cells harboring pG8_crRNA transformed with compatible plasmid containing protospacer. Individual transformants were grown at conditions of induction of *cas* genes in the absence of ampicillin in the medium, thus allowing the protospacer plasmid loss. After overnight growth, culture aliquots were analyzed by PCR to monitor CRISPR array expansion. KD263 cells transformed with protospacer plasmid were used as a control. The results are presented in Figure 6A. As expected, adaptation was observed in KD263 cells (18,32). Similar levels of adaptation were also observed in KD390 and H20A cells transformed with protospacer plasmid. A somewhat reduced adaptation level was detected in Δcas6e cultures. No adaptation was observed in cells harboring a plasmid without a protospacer. Therefore, unit-sized crRNA generated without Cas6e processing appears to support primed adaptation and the endoribonucleolytic activity of Cas6e (and in fact the Cas6e protein itself) is dispensable in this context.

PCR-amplified fragments corresponding to spacer acquisition events in cultures of KD263, KD390, H20A and Δcas6e were subjected to Illumina sequencing (32). Spacer sequences were derived from individual filtered reads and mapped on the protospacer plasmid. The statistics of this analysis is presented in Figure 6B. As can be seen, the preference for AAG PAMs in protospacers used as donors of newly acquired spacers remained high (and characteristic of primed adaptation) in all four samples, gradually decreasing from 94 to 90% in the order: KD263>KD390>H20A>Δcas6e. The strand bias of protospacer selection also decreased significantly in the order: KD263>KD390>H20A>Δcas6e, from 90% in KD263, a typical value for primed adaption (18,32), to 72% in Δcas6e.

A significant percentage of spacers originating from a given protospacer can be inserted in CRISPR cassette in an ‘inverted’ orientation, thus affecting the apparent strand bias of spacer acquisition (33). In addition, some acquired spacers are shifted, being offset with respect to correct PAM by one or several bases either in upstream or downstream direction and leading to apparent decrease of preference for an AAG PAM (33). The total number of such ‘derived’, inverted and shifted, spacers was twice higher in Δcas6e cells than in KD263 (7.7 versus 3.9% of the total number of acquired spacers). When ‘derived’ spacers were considered as originating from correct primary protospacer recognition events, the preference for AAG PAM reached 98% in all samples (Figure 6B). However, decreased strand bias in cells harboring crRNA generated by transcription termination and, especially, with mutant Cas6e or lacking Cas6e altogether was still apparent, indicating that some other mechanism is responsible for the observed increased percentage of spacers chosen from the ‘wrong’ strand during primed adaptation in these cells.

Earlier, we showed that some spacers are reproducibly acquired much more efficiently than others, presumably because certain protospacers are better recognized by the acquisition machinery (32). The distribution of spacers acquired in four different cells is shown in Figure 6C. Apart from the already mentioned increased tendency to acquire spacers from the ‘wrong’, targeted strand in H20A cells and, especially, in Δcas6e cells, the overall distribution and usage efficiency of protospacer donors was very similar in all cases. Pearson correlation coefficients between samples were 0.9 and higher, indicating that neither Cas6e nor the mechanism by which mature crRNA is generated influence the efficiency of donor protospacer choice.

## DISCUSSION

In this paper, we show that the *E. coli* subtype I-E CRISPR-Cas system can interfere with bacteriophage infection and plasmid transformation and perform primed acquisition of new spacers in the absence of Cas6e activity and that partial interference with plasmid transformation and efficient primed adaptation can even occur in the absence of Cas6e. Cas6e is a component of the Cascade complex, an endoribonuclease that generates unit-sized crRNA from pre-crRNA precursor transcript and is normally essential for CRISPR-Cas function. We show that Cas6e becomes redundant when unit-sized crRNA is generated by RNA polymerase through factor-independent transcription termination on an artificial terminator based on CRISPR repeat, located 3′ terminal to the guide segment of crRNA transcription unit. While our work was in progress, the Marchfelder group demonstrated that subtype I-B crRNA matured in Cas6b-independent way in *Haloferax* cells is active in CRISPR interference (22). Moreover, they showed that when crRNA was provided independently of Cas6b by excision from a pre-crRNA using tRNA processing enzymes, the Cas6b protein became dispensable for interference. Together, the results suggest that Cas6 is only essential function in all Type I systems, may be limited to generation of processed crRNA. In *E. coli*, the Cas6e protein also appears to play a role in stabilizing crRNA interaction with Cascade, likely through participating in simultaneous interactions with the 3′ repeat handle of crRNA and subunits of Cascade. While contributing to overall robustness of CRISPR interference, this role is secondary and both CRISPR interference and adaption can occur in the absence of Cas6e.

As follows from the acronym ‘CRISPR’, most repeats (from Type I and Type III systems) are palindromic. This property is essential for pre-crRNA processing into crRNAs, however, it should also create a potential problem during transcription of the array, for palindromic sequences should create multiple transcription elongation pause sites as well as transcription terminators. To our knowledge, our results demonstrate for the first time that a CRISPR repeat can function as a highly efficient transcription terminator when followed by a T-tract. Apart from the practical use of this finding described in our paper, the results suggest that transcription of long untranslated CRISPR arrays may have to be assisted by yet to be identified transcription elongation factors, similar to the situation observed during transcription of rRNA genes (34). Alternatively, Cas6 proteins can bind to hairpin structures formed in nascent pre-crRNA transcripts and thus ensure the processivity of transcription. The potential of CRISPR repeats to stall elongation of transcription may be responsible for decreased expression of promoter-distal crRNAs and may also provide counter selection against A/T rich spacers, which should negatively regulate expression of downstream crRNAs.

After processing pre-crRNA, Cas6e remains tightly bound to the 3′-handle of crRNA. Since Cas6e is dispensable for crRNA binding to Cascade and target recognition, it follows that the 3′-handle of crRNA should also be dispensable. This is indeed what was observed in the context of complete Cascade (23) and we here show that removal of both the 3′ handle and Cas6e does not affect target recognition and R-loop formation, at least *in vitro*. Results obtained in the Haloferax system (22) strongly suggest that this is also the case *in vivo*. Thus, the Cas6e(Cas6b)-crRNA 3′ handle interaction plays only a limited role in CRISPR response in their respective systems, namely generating unit-sized crRNA from the initial CRISPR array transcript and stabilizing the interference complex.

In subtype I-E system, the minimal assembly capable of target recognition contains a Cascade lacking Cas6e (Cascade^Cse1Cse2Cas5Cas7^) and a crRNA containing the 5′ repeat handle, the spacer, and just one nucleotide of the 3′ repeat handle. One can speculate that involvement of a pre-crRNA endonuclease generating unit-sized crRNAs became necessary only at late stages of evolution of CRISPR-Cas systems, when ancestral CRISPR arrays containing just one spacer started to expand after spacer acquisition machinery (Cas1/Cas2) became appropriated by ancient interference systems. This has been achieved through radically different ways in type II systems (through involvement of non-coding tracrRNA and host RNase, (35)) or dedicated pre-crRNA nucleases specifically recognizing repeats (in type I and type III systems). It is interesting that in organisms containing both type III systems subtypes, IIIA and IIIB, the same protein, Cas6, simultaneously serves different type IIIA and type IIIB effector complexes (36), providing them with identical crRNAs that can then target either DNA or RNA (type IIIA) or RNA (type IIIB). Such sharing between different systems is also consistent with late arising of pre-crRNA processing enzymes.

Inspection of Cascade preparations purified from cells lacking Cas6e by native PAGE reveals multiple protein complexes, which are absent from complete Cascade samples and are likely formed due to oligomerization of Cas7 beyond the hexamer present in functional Cascade (Supplementary Figure S6). Thus Cas6e may also play a structural role by limiting Cas7 oligomerization and ensuring that only crRNAs with correct spacer length are bound to the effector complex. It is conceivable that Cascade species lacking Cas6e and containing extended or shortened Cas7 oligomers will be able to interact with crRNAs with appropriately altered guide segment length and then recognize the correspondingly altered targets. Indeed, archaeal type III-B CRISPR-Cas Cmr complex, which is a functional analog of Cascade, when programmed with shortened or lengthened crRNAs was able to cleave some targets with higher efficiency than the wild-type crRNA-Cmr complex, possibly due to changes in stoichiometry of oligomer formed by Cmr4, a Cas7 analog (37). Experiments aimed to prove this conjecture in the case of *E. coli* Cascade are currently underway.

The finding that unit-sized crRNA produced by transcription termination is functional in both CRISPR interference and adaptation greatly simplifies structure-activity analysis of *E. coli* crRNA. Previously, strains for such experiments had to be constructed by complex engineering of genomic or plasmid borne CRISPR arrays (5,9,18) and depended on proper processing of pre-crRNA (5,9). Availability of functional unit-sized crRNAs generated by RNA polymerase *in vivo* overcomes this limitation, making it possible to rapidly introduce mutations in either repeat or guide segments of crRNA.

The ability to rapidly generate crRNAs with various guide sequences from plasmid-borne unit-sized transcription units allows one to efficiently interrogate any host or bacteriophage/plasmid target of interest followed by a simple readout using cell viability, phage infection and/or plasmid transformation interference assays. This should help to determine how CRISPR-Cas immunity affects mobile genetic elements that rely on different strategies to take over the bacterial host. When Cas6e endonuclease is inactivated, unit-length crRNA transcripts generated by transcription termination and containing additional, non-repeat derived sequences enter Cascade and function *in vivo*. This observation indicates that similarly to type II CRISPR-Cas systems (38), engineering of type I crRNA that endow it with new functionalities and ability to engage additional factors to sites recognized by crRNA guide sequence should be possible, adding further versatility to this system.

## SUPPLEMENTARY DATA

Supplementary Data are available at NAR Online.

SUPPLEMENTARY DATA
