# Supplementary material for: The Cas6e ribonuclease is not required for interference and adaptation by the E. coli type I-E CRISPR-Cas system
Source: Nucleic Acids Res. 2015 May 26;43(12):6049–61. doi: 10.1093/nar/gkv546 (PMC4499155; doi:10.1093/nar/gkv546)
Supplement: SUPPLEMENTARY DATA [file supp_43_12_6049__index.html]

The Cas6e ribonuclease is not required for interference and adaptation by the E. coli type I-E CRISPR-Cas system — SUPPLEMENTARY DATA 

# The Cas6e ribonuclease is not required for interference and adaptation by the *E. coli* type I-E CRISPR-Cas system

## SUPPLEMENTARY DATA

- SUPPLEMENTARY DATA
