# Supplementary material for: The Cas6e ribonuclease is not required for interference and adaptation by the E. coli type I-E CRISPR-Cas system
Source: Nucleic Acids Res. 2015 May 26;43(12):6049–61. doi: 10.1093/nar/gkv546 (PMC4499155; doi:10.1093/nar/gkv546)
Supplement: SUPPLEMENTARY DATA [file supp_gkv546_nar-00631-h-2015-File002.doc]

**Supplementary materials**

**Table S1.** Plasmids used in this study

| **Plasmids** | **Description** | **Reference** |
| --- | --- | --- |
| pG8_dir | g8 protospacer blunt-end-cloned in pT7Blue | (1) |
| pG8_dir_C1T | g8 protospacer with C1T mutation blunt-end-cloned in pT7Blue | (1) |
| pWUR615 | *E. coli* CRISPR, 7 x spacer g8, in pACYCDuet-1 (Cm^R^) | (2) |
| pG8_crRNA | pACYC (Cm^R^) producing g8-crRNA under T7A1 promoter^1^ | This study |
| pG8trp_crRNA | pACYC (Cm^R^) producing g8-crRNA under trp promoter^2^ | This study, (3) |
| pSpT5_crRNA | pACYC (Cm^R^) producing SpT5-crRNA under T7A1 promoter^3^ | This study |
| pSpλ_crRNA | pACYC (Cm^R^) producing Spλ-crRNA under T7A1 promoter^4^ | This study |
| pG8_crRNA _C1T | pG8_crRNA with C1T mutation in g8 spacer^5^ | This study |
| pCDF-casABCDE | *cse1-cse2-cas7-cas5-cas6e* in pCDF-1b (Sm^R^), *cse2* with N-terminal StrepII-tag | (4) |
| pCDF-casBCDE | *cse1* was deleted from pCDF-casABCDE | This study |
| pCDF-casBCDE**^H20A^** | pCDF-casBCDE with H20A mutation in Cas6e | This study |
| pCDF-casBCD | *cas6e* was deleted from pCDF-casBCDE | This study |
| p15-casABCDE | *cse1-cse2-cas7-cas5-cas6e* in p15Tv-L (Ap^R^): *cse1* with N-terminal 6His-tag | (4) |
| p15-casABCD | *cas6e* was deleted from p15-casABCDE | This study |

**Synthetic gBlocks sequences used for crRNA-plasmid construction** (crRNA-coding region is underlined with spacer sequence highlighted in bold type, the -35 and -10 promoter regions are shown in bold type, *Eco*NI and *Kpn*I cloning sites are shown in italic type)

^1^pG8_crRNA:

GTCGAT*CCTGCATTAGG*AAAGAGTA**TTGACT**TAAAGTCTAACCTATAG**GATACT**TACAGCCATAAACCG**CTGTCTTTCGCTGCTGAGGGTGACGATCCCGC**GAGTTCCCCGCGCCAGCGGGGATTTTTTTTATCCATATGACTAGTAGATCCTCT*GGTACC*AGCTGA

^2^pG8trp_crRNA:

GTCGAT*CCTGCATTAGG*ACATCATAACGGTTCTGGCAAATATTCTGAAATGAGCTG**TTGACA**ATTAATCATCGAACTAG**TTAACT**AGTACGCATAAACCG**CTGTCTTTCGCTGCTGAGGGTGACGATCCCGC**GAGTTCCCCGCGCCAGCGGGGATTTTTTTTATCCATATGACTAGTAGATCCTCT*GGTACC*AGCTGA

^3^pSpT5_crRNA:

GTCGAT*CCTGCATTAGG*AAAGAGTA**TTGACT**TAAAGTCTAACCTATAG**GATACT**TACAGCCATAAACCG**AAATTGGGCAAATTAAACACCGGACACACCCA**GAGTTCCCCGCGCCAGCGGGGATTTTTTTTATCCATATGACTAGTAGATCCTCT*GGTACC*AGCTGA

^4^pSpλ_crRNA :

GTCGAT*CCTGCATTAGG*AAAGAGTA**TTGACT**TAAAGTCTAACCTATAG**GATACT**TACAGCCATAAACCG**ATAACGCTTGTGAAAATGCTGAATTTCGCGTC**GAGTTCCCCGCGCCAGCGGGGATTTTTTTTATCCATATGACTAGTAGATCCTCT*GGTACC*AGCTGA

^5^pG8_crRNA_C1T

Primers used for mutagenesis:

5’ – TACAGCCATAAACCG**T**TGTCTTTCGCTGCTG – 3’

5’ – CAGCAGCGAAAGACA**A**CGGTTTATGGCTGTA – 3’

**Table S2.** Strains used in this study

| **Strains** | **Description** | **Reference** |
| --- | --- | --- |
| KD418 | BL21 Star (DE3) with CRISPR1 deleted | (4) |
| KD263 | K12 F’, *lac*UV5-*cas3 araBp*8-*cse1,* Repeat-g8 spacer-Repeat (CRISPR I), ΔCRISPR II +III | (5) |
| KD390 | K12 F’, *lac*UV5-*cas3 araBp*8-*cse1,* Repeat (CRISPR I), ΔCRISPR II +III | This study |
| KD477 | K12 F’, *lac*UV5-*cas3 araBp*8-*cse1,* with H20A mutation in Cas6e, Repeat (CRISPR I),  ΔCRISPR II +III | This study |
| KD599 | K12 F’, *lac*UV5-*cas3 araBp*8-*cse1,* with *cas6e* deleted, Repeat (CRISPR I), ΔCRISPR II +III | This study |

**Figure S1. Generation of unit-size** **g8-crRNA by transcription termination during transcription *in vitro*.** A template for *in vitro* transcription was obtained by PCR amplification of a fragment of plasmid pG8_crRNA; the amplified fragment boundaries were -96/+149, with transcription start site located at +1. The fragment was transcribed with *E. coli* RNAP σ^70^ holoenzyme as described in Experimental Procedures. Positions of the run-off (RO) and terminated transcripts (lane 1, panel A) are indicated by arrowheads on the left and the structure of terminated transcript is schematically shown. As molecular markers, 5’-P^32^ labeled synthetic crRNA with the size of 61 nt (lane 3, panel A) and 53-nt crRNA derivative lacking the 5’ end-proximal repeat sequences (4) (lane 2, panel A) were used. Panel B shows a typical example of *in vitro* transcription from the T7 A1 promoter fused to a natural terminator λ tR2.


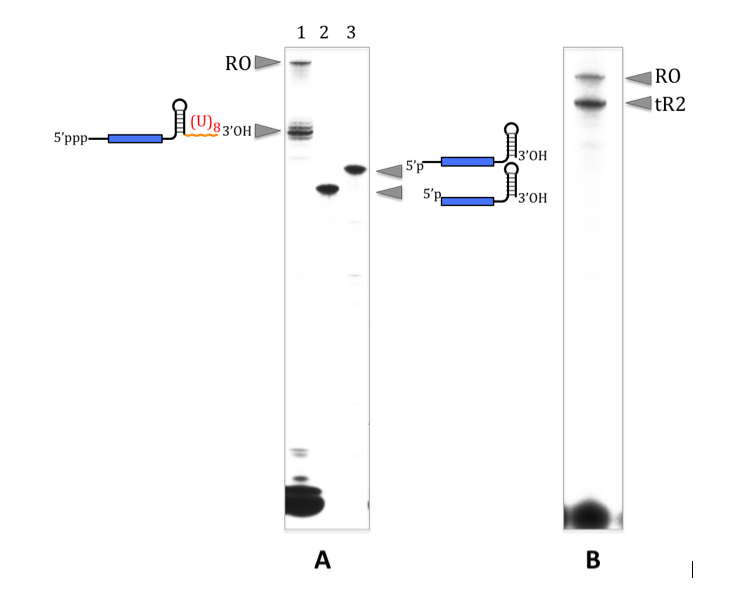


**Figure S2.** A Northern blot showing the results of hybridization of total RNA purified from KD263 cells (lane 1 in Fig. 1C) along with Decade RNA size markers (sizes, in nucleotides, are shown on the left).


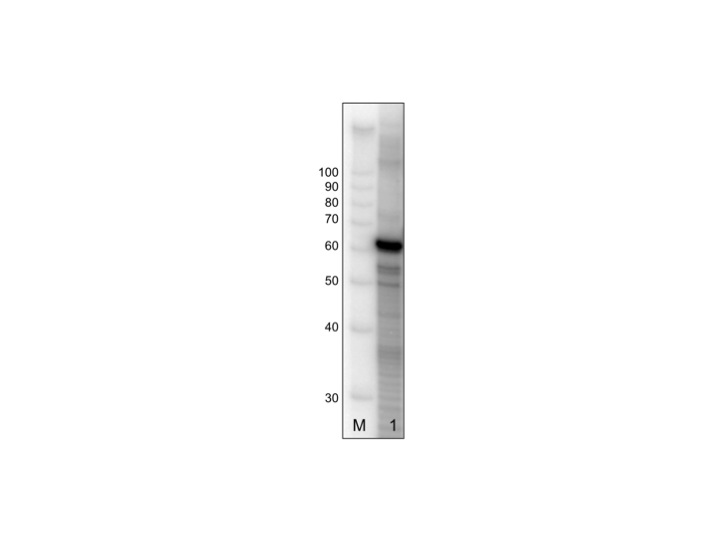


**Figure S3.** RNA was extracted from Cascade preparations (shown in Fig. 1C, lanes 6, 7, and 8) and radioactively labeled with T4 nucleotide kinase. As a control, chemically synthesized 61-nt RNA oligonucleotide matching mature g8 crRNA was used (RNA_61_). Samples were resolved by denaturing PAGE and revealed by autoradioagraphy.


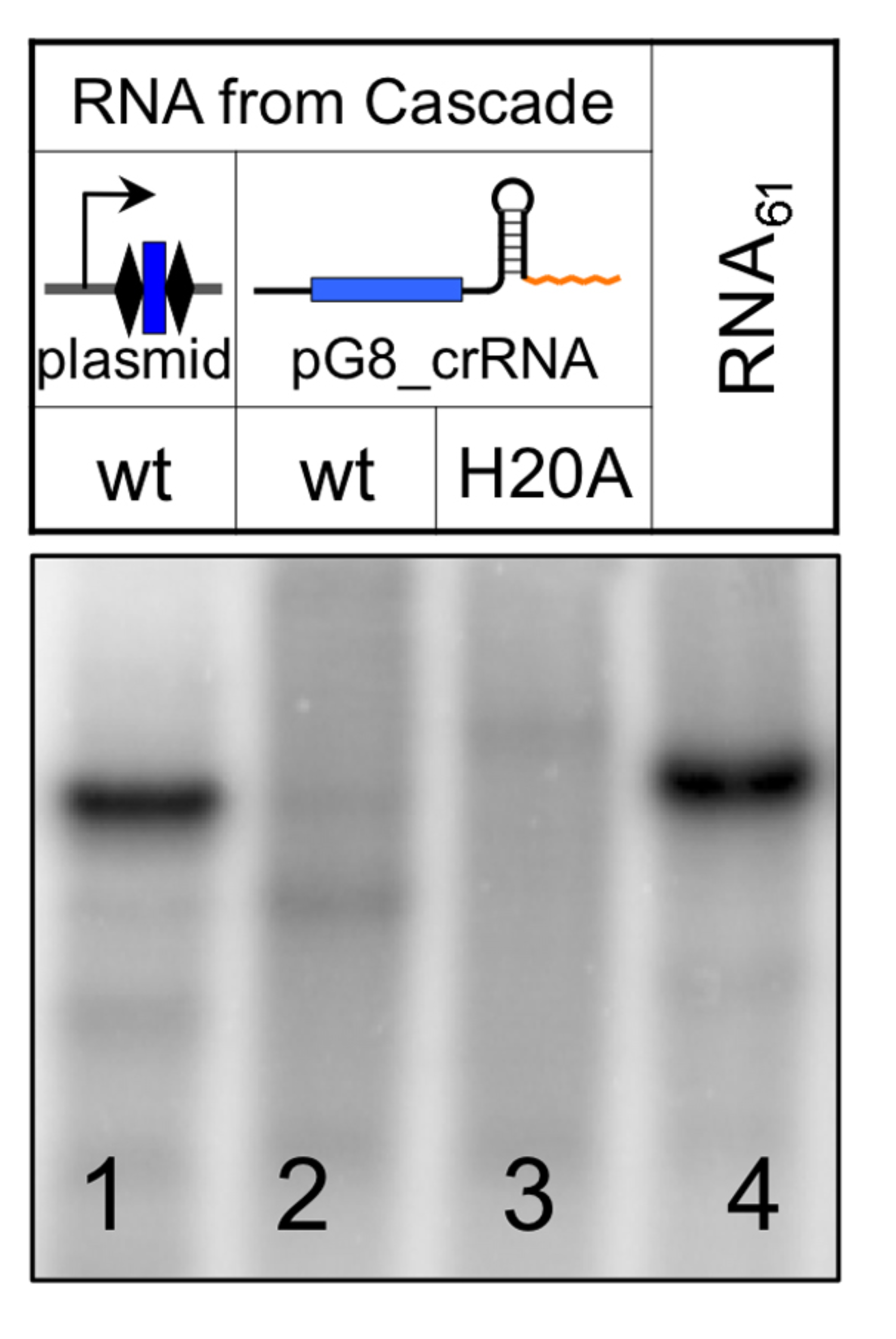


**Figure S4.** SDS-PAGE analysis of Cascade preparation used in Fig. 5A. A Coomassie-stained gel is shown. Asterisk indicates Strep-tagged Cse2 used for affinity purification.


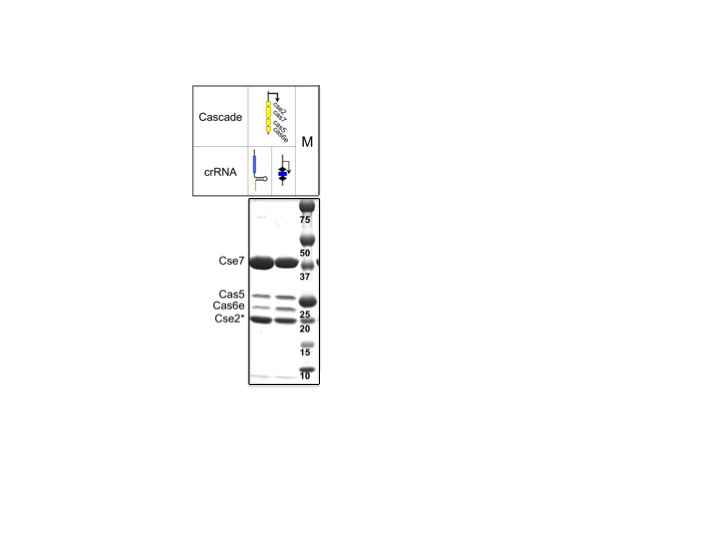


**Figure S5.** SDS-PAGE analysis of Cascade preparation used in Fig. 5B. A Coomassie-stained gel is shown. Asterisk indicates His-tagged Cse1 used for affinity purification. After affinity purification samples were further purified by gel filtration.

**
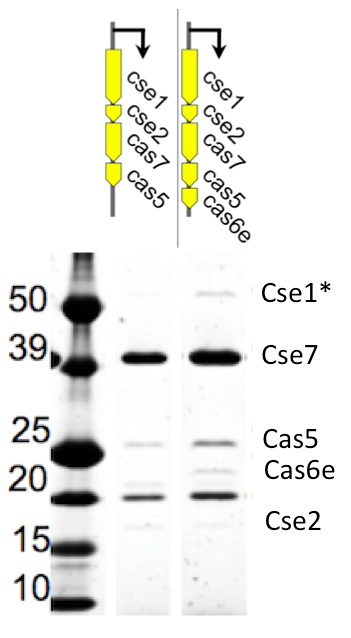
**

**Figure S6.** A native-PAGE analysis of Cascade preparation with and without Cas6e. A Coomasie-stained gel is shown.


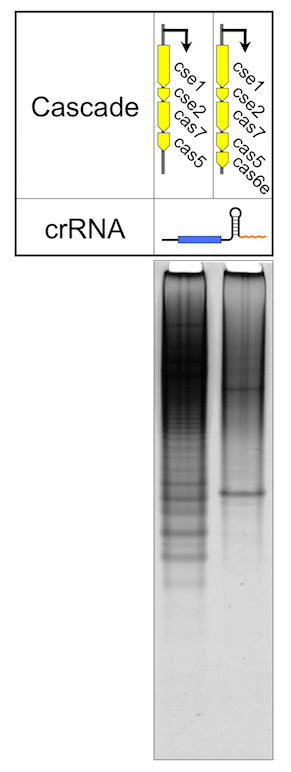


**SUPPLEMENTARY REFFERANCES**

1. Datsenko, K. A., Pougach, K., Tikhonov, A., Wanner, B. L., Severinov, K. and Semenova, E. (2012) Prior encounters dramatically stimulate adaptive bacterial CRISPR immune response to viruses. *Nature Commun.,* **3,** 945.
2. Semenova, E., Jore, M.M., Datsenko, K.A., Semenova, A., Westra, E.R., Wanner, B., van der Oost, J., Brouns, S.J. and Severinov, K. (2011) Interference by clustered regularly interspaced short palindromic repeat (CRISPR) RNA is governed by a seed sequence. *Proc. Natl. Acad. Sci. USA*, **108**, 10098-10103.
3. Bass, S.H. and Yansura, D.G. (2000) Application of the E. coli trp promoter. *Mol Biotechnol*, **16**, 253-260.
4. Beloglazova, N., Kuznedelov, K., Flick, R., Datsenko, K. A., Brown, G., Popovic, A., Lemak, S., Semenova, E., Severinov, K. and Yakunin, A. F. (2015) CRISPR RNA binding and DNA targeting by purified Cascade complexes from *Escherichia coli*. *Nucleic Acids Res.,* **43,** 530-543.
5. Shmakov, S., Savitskaya, E., Semenova, E., Datsenko, K. A. and Severinov, K. (2014) Pervasive generation of oppositely-oriented spacers during CRISPR adaptation. *Nucleic Acids Res.,* **42,** 5907-5916.
